# Cytotoxicity of 11-*epi*-Sinulariolide Acetate Isolated from Cultured Soft Corals on HA22T Cells through the Endoplasmic Reticulum Stress Pathway and Mitochondrial Dysfunction

**DOI:** 10.3390/ijms17111787

**Published:** 2016-10-27

**Authors:** Jen-Jie Lin, Robert Y. L. Wang, Jiing-Chuan Chen, Chien-Chih Chiu, Ming-Hui Liao, Yu-Jen Wu

**Affiliations:** 1 Graduate Institute of Veterinary Medicine, National Pingtung University of Science and Technology, Pingtung 91201, Taiwan; q87634@hotmail.com; 2 Department of Biomedical Sciences and Molecular Medicine Research Center, College of Medicine, Chang Gung University, Taoyuan 33302, Taiwan; yuwang@mail.cgu.edu.tw; 3 Division of Pediatric Infectious Disease, Department of Pediatrics, Chang Gung Memorial Hospital, Linkuo 33305, Taiwan; 4 Department of Food Science and Nutrition, Meiho University, Pingtung 91202, Taiwan; x00000017@meiho.edu.tw; 5 Department of Biotechnology, Kaohsiung Medical University, Kaohsiung 80708, Taiwan; woodnettle2002@gmail.com; 6 Department of Beauty Science, Meiho University, Pingtung 91202, Taiwan

**Keywords:** 11-*epi*-sinulariolide acetate, mitochondrial dysfunction, ER stress, antitumor, hepatocellular carcinoma

## Abstract

Natural compounds from soft corals have been increasingly used for their antitumor therapeutic properties. This study examined 11-*epi*-sinulariolide acetate (11-*epi*-SA), an active compound isolated from the cultured soft coral *Sinularia flexibilis*, to determine its potential antitumor effect on four hepatocellular carcinoma cell lines. Cell viability was investigated using 3-(4,5-dimethylthiazol-2-yl)-2,5-diphenyltetrazolium bromide assay, and the results demonstrated that 11-*epi*-SA treatment showed more cytotoxic effect toward HA22T cells. Protein profiling of the 11-*epi*-SA-treated HA22T cells revealed substantial protein alterations associated with stress response and protein synthesis and folding, suggesting that the mitochondria and endoplasmic reticulum (ER) play roles in 11-*epi*-SA-initiated apoptosis. Moreover, 11-*epi*-SA activated caspase-dependent apoptotic cell death, suggesting that mitochondria-related apoptosis genes were involved in programmed cell death. The unfolded protein response signaling pathway-related proteins were also activated on 11-*epi*-SA treatment, and these changes were accompanied by the upregulated expression of *growth arrest and DNA damage-inducible protein* (*GADD153*) and *CCAAT/enhancer binding protein* (*C/EBP*) *homologous protein* (*CHOP*), the genes encoding transcription factors associated with growth arrest and apoptosis under prolonged ER stress. Two inhibitors, namely salubrinal (Sal) and SP600125, partially abrogated 11-*epi*-SA-related cell death, implying that the protein kinase R (PKR)-like endoplasmic reticulum kinase (PERK)–activating transcription factor (ATF) 6–CHOP or the inositol-requiring enzyme 1 alpha (IRE1α)–c-Jun N-terminal kinase (JNK)–cJun signal pathway was activated after 11-*epi*-SA treatment. In general, these results suggest that 11-*epi*-SA exerts cytotoxic effects on HA22T cells through mitochondrial dysfunction and ER stress cell death pathways.

## 1. Introduction

Hepatocellular carcinoma (HCC), or primary liver cancer, one of the most aggressive and common malignancies, is the third highest cause of cancer-related mortality worldwide, and accounts for 75%–85% of the primary malignant tumors in the liver [1,2]. According to a medical report, HCC is an aggressive malignancy with a median post-diagnosis survival period of approximately 6–20 months [3]. In addition, the five-year survival rate of HCC is lower than 50% [4,5]. Although surgery is the major effective therapeutic approach for treating patients with HCC, only 20%–30% of patients are eligible for surgical resection. By contrast, a high dose of chemotherapy or radiotherapy is required for most HCC patients, but the side effects of these modalities are excessively severe to tolerate [6,7]. Therefore, exploring more effective and natural compounds for treating HCC is necessary.

Nearly a decade ago, several new therapeutic applications of natural products from soft corals were widely investigated for treating HCC [8,9,10,11]. Chemical compounds, such as diterpenoids, diterpenes, and prostanoids, isolated from marine soft corals, exert various biological activities, including antiproliferation, antimigratory, and antiapoptotic, on different cancer cell lines, such as prostate cancer cells, HCC cells, breast cancer cells, colon cancer cells, cervical cancer cells, oral squamous cells, bladder cancer cells, and melanoma cells [12,13,14,15,16,17].

The apoptosis-induced activity of anticancer therapies is a valuable guide for predicting tumor response after anticancer treatment. Cell apoptosis causes cell shrinkage, chromatin condensation, nucleus fragmentation, and apoptosome complex formation. Cell apoptosis naturally occurs in cells and may enhance the development of cancer therapy [18]. Apoptosis, a proteolytic process, involves a family of caspases, which are characteristic of the apoptotic process. Caspases indicated as proapoptotic signal initiators are caspase 8, 9, and 10, which initiate a proteolytic cascade. These initiators, in turn, activate the effector caspase 3, 6, and 7, which amplify the signal by cleaving initiator caspases, leading to morphological and biochemical changes that are representative of apoptosis [19,20,21,22]. The B-cell lymphoma 2 (Bcl-2) protein family contains apoptosis regulators: proapoptotic members, including the multidomain Bcl-2-associated X (Bax) and Bcl-2 antagonist/killer 1 (Bak) proteins; different single-domain BH3-only proteins Bcl-2-associated death promoter (Bad), Bcl-2 interacting mediator of cell death (Bim), BH3 interacting-domain death agonist (Bid), and p53-upregulated modulator of apoptosis (PUMA); and antiapoptotic members, such as Bcl-2, B-cell lymphoma-extra large (Bcl-x_L_), and myeloid cell leukemia-1 (Mcl-1) [23].

Anticancer therapies mainly inhibit cancer cells through apoptotic processes, which can be triggered through the extrinsic and intrinsic pathways [24,25,26]. Recent studies have implied that the initiation of intrinsic pathways may be associated with mitochondrial and endoplasmic reticulum (ER) stress [26,27,28]. Mitochondrial dysfunction is a major incident during cellular apoptosis. Bax protein activation seems to play a central role in mitochondria-dependent apoptosis. During apoptosis, Bax translocates into the mitochondrial outer membrane where it becomes activated and forms pores through oligomerization that trigger mitochondrial dysfunction [29,30,31]. An increased ratio of Bax/Bcl-2 stimulates cytochrome *c* release from the mitochondrial intermembrane space into the cytosol, where cytochrome *c* can bind to A-Raf-1, leading to the activation of caspase-3 [32,33,34,35]. Caspase-3 is displayed to cleave its substrate poly(ADP-ribose) polymerase 1 (PARP-1) to induce characteristic apoptotic changes, such as chromatin condensation and DNA fragmentation [36].

The ER is a critical location for regulating protein folding, protein synthesis, and intracellular Ca^2+^ homeostasis [37]. Unfolded protein response (UPR) is triggered in the ER for releasing ER stress, and it certainly causes apoptosis if the ER stress becomes prolonged and severe [38]. Activating UPR essentially requires glucose-regulated protein 78 (GRP78)/ binding immunoglobulin protein (Bip), an ER chaperone, and a regulator of three ER sensors, PERK (protein kinase R RNA (PKR)- like ER kinase), IRE1 (inositol-requiring enzyme 1) and ATF6 (activating transcription factor-6) [39].

Therefore, treating HCC is a crucial medical and therapeutic concern. Exploring new effective anticancer drugs and developing anti-HCC therapies are necessary. Previous studies have demonstrated that 11-*epi*-SA, an active compound isolated from the cultured soft coral *Sinularia flexibilis*, inhibits *cyclooxygenase-2* and *interleukin-8* gene expression, and suppresses inflammatory responses and bone destruction in adjuvant-induced arthritis [40,41]. Here, we applied a comparative proteomics approach to describe the possible molecular mechanism of the apoptosis-induced and other antitumor effects of 11-*epi*-SA on HA22T HCC cells. Differential expression of proteins was identified through two-dimensional gel electrophoresis (2DE) and liquid chromatography–tandem mass spectrometry (LC–MS/MS). In combination with other biochemical characterizations, we demonstrated that 11-*epi*-SA induces cell apoptosis through at least the mitochondrial dysfunction and ER stress pathways.

## 2. Results

### 2.1. 11-epi-SA Is Cytotoxic toward Several HCC Cell Lines

**Figure 1:**
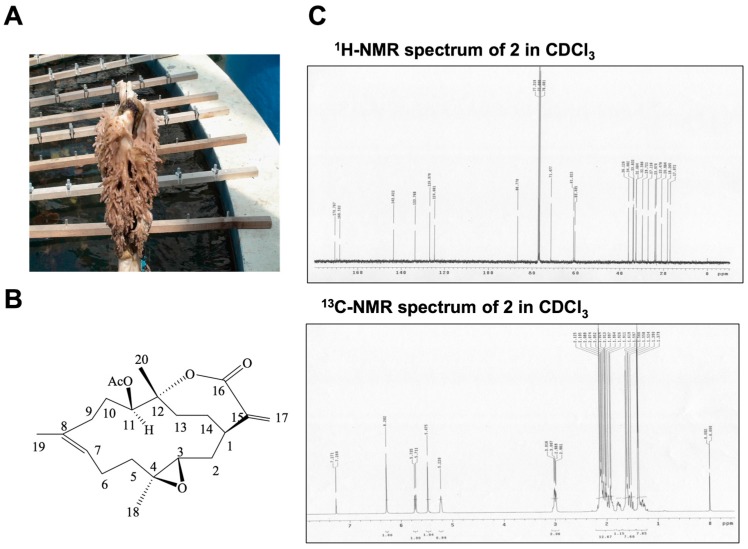
General characteristics of 11-*epi*-sinulariolide acetate (11-*epi*-SA). (**A**) Photograph of cultured soft coral *Sinularia flexibilis* obtained from the National Museum of Marine Biology and Aquarium, Pingtung, Taiwan; (**B**) Chemical structure of 11-*epi*-SA. The molecular formula and molecular weight of 11-*epi*-SA are C_22_H_32_O_5_ and 376 Da, respectively; (**C**) Typical proton nuclear magnetic resonance (^1^H-NMR) spectrum of 11-*epi*-SA in deuterochloroform (CDCl_3_) at 400 MHz and carbon-13 (^13^C)-NMR spectrum of 11-*epi*-SA in CDCl_3_ at 100 MHz.

**Figure 2:**
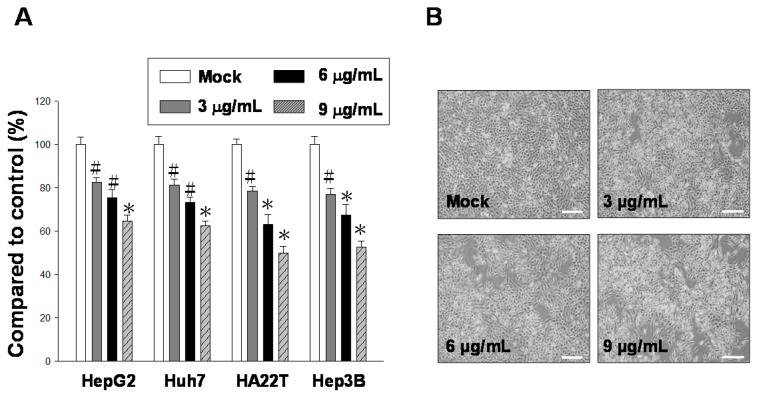
Administering 11-*epi*-SA treatment to HA22T hepatoma cells causes cytotoxic effects. (**A**) Investigation of the cytotoxic effect of 11-*epi*-SA on human hepatoma cell lines. Four human hepatoma cell lines (HepG2, Huh7, HA22T, and Hep3B) were treated with dimethyl sulfoxide (DMSO) (mock control) or various concentrations of 11-*epi*-SA for 24 h, followed by the examination of cell viabilities by using the 3-(4,5-dimethylthiazol-2-yl)-2,5-diphenyltetrazolium bromide (MTT) assay as described in the Materials and Methods section. The DMSO-treated cells exhibited 100% viability. 11-*epi*-SA exerts a considerable cytotoxic effect on HA22T cells. The results shown here are representative of three independent experiments (# *p* < 0.05; * *p* < 0.01); (**B**) Morphological changes of the HA22T cells on 11-*epi*-SA treatment. HA22T cells were treated with DMSO (Mock) or 11-*epi*-SA at final concentrations of 3, 6, and 9 μg/mL. Scale bar: 50 µm.

Active compounds from natural products have been used for antitumor therapy because such compounds exhibit a cytotoxic effect on cancer cells [16,42]. In this study, the main chemical component 11-*epi*-SA was isolated from the cultured soft coral *S. flexibilis* (Figure 1). To explore the potential antitumor effect of 11-*epi*-SA on HCC cells, we first examined the cytotoxic effects of 11-*epi*-SA on four different HCC lines, namely HepG2, Huh7, HA22T, and Hep3B cells. Three concentrations (3, 6, and 9 μg/mL) of 11-*epi*-SA were used for treating these cell lines for 24 h, after which cell viability was investigated using the 3-(4,5-dimethylthiazol-2-yl)-2,5-diphenyltetrazolium bromide (MTT) assay. The results revealed that the HA22T cells were more sensitive to the 24 h 11-*epi*-SA treatment compared with the other cells (Figure 2A). Compared with the control cells, approximately 54% of HA22T cells exposed to 9 μg/mL 11-*epi*-SA were viable, indicating the cytotoxic effect of 11-*epi*-SA on the HA22T cells. Because the HA22T cell line was more sensitive to 11-*epi-*SA treatment, we investigated the anticancer effect of 11-*epi*-SA on the HA22T cells. First, the morphological changes of the 11-*epi*-SA-treated cells were investigated through inverted light microscopy. Compared with the dimethyl sulfoxide (DMSO)-treated (Mock control) cells, the 11-*epi*-SA-treated HA22T cells exhibited shrinkage as well as decreased cell population (Figure 2B). By contrast, we treated other cell lines (HaCat and Hs68) with 11-*epi-*SA to test its cytotoxicity. There was no cytotoxicity effect of 11-*epi*-SA (even at the high dosage of 12 μg/mL) neither on HaCat nor Hs68 cells (Figure S1), indicating that this natural compound may exhibit a specific effect towards human hepatoma cells. Also, the range of 11-*epi*-SA concentrations used did not appear to be cytotoxic to any of the four cell lines. Altogether, these results demonstrate the antiproliferative effect of 11-*epi*-SA on HA22T cells.

**Figure 3:**
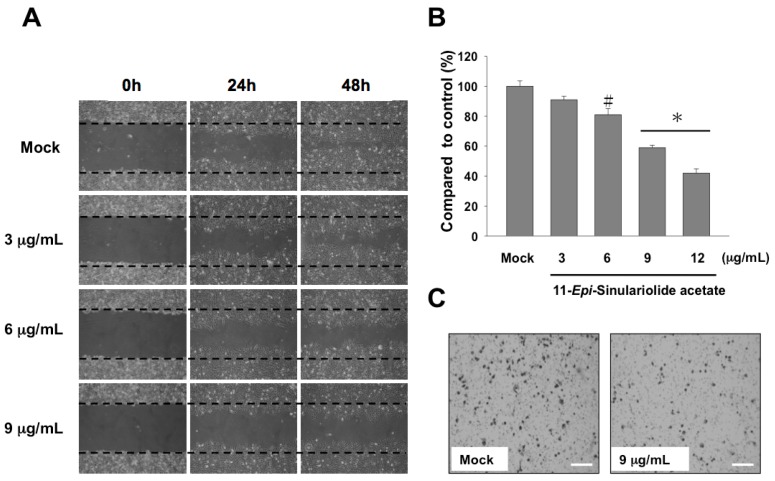
Treatment with 11-*epi*-SA inhibits cell migration and induces the appearance of apoptotic characteristics in HA22T cells. (**A**) Mock- or 11-*epi*-SA-treated HA22T cells in the areas between two dotted lines are cells that have migrated during the indicated time periods. Images represent the apparent reduction of HA22T cell migration after treatment with 3, 6, and 9 μg/mL 11-*epi*-SA for 24 and 48 h, respectively; (**B**) Quantitative measurement of the migration of the HA22T cells is shown and compared with that of the DMSO-treated cells. The result shows dose-dependent suppression of HA22T cell migration (# *p* < 0.05; * *p* < 0.001). Data shown are representative of three independent experiments; (**C**) Enlarged view showing reduced migration of the HA22T cells (after treatment with 9 μg/mL 11-*epi*-SA) compared with the DMSO-treated cells at 100× magnification. Scale bar: 50 µm.

Second, the migration capacity of the 11-*epi*-SA-treated HA22T cells was assessed using wound healing and cell migration assays. After the 24 and 48 h treatment with 11-*epi*-SA (at final concentrations of 3, 6, and 9 μg/mL), the wound healing assay revealed that the migration of the 11-*epi*-SA-treated cells was inhibited (Figure 3A). The quantitative data of the cell migration assay showed a dose-dependent suppression on 11-*epi*-SA treatment at the concentrations of 3–9 μg/mL. The suppression rates were approximately 18% and 41% at the concentrations of 6 and 9 μg/mL of 11-*epi*-SA that led in suppression of HA22T cell growth (Figure 3B,C). Overall, these results clearly demonstrated that 11-*epi*-SA exerted a cytotoxic effect on the HA22T cells.

### 2.2. HA22T Cells Show Apoptotic Characteristics on 11-epi-SA Treatment

**Figure 4:**
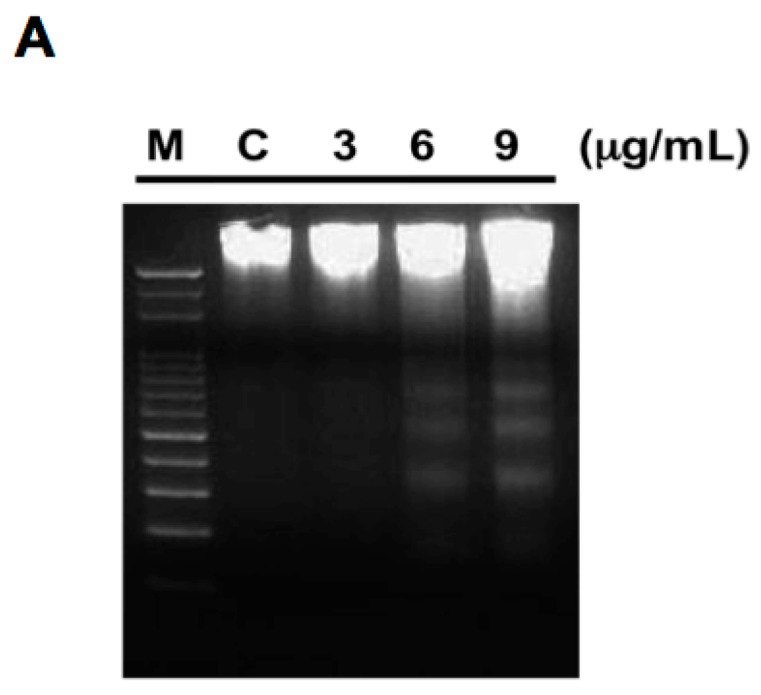

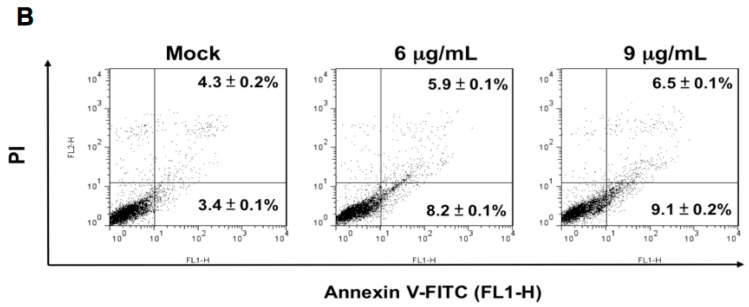
Appearance of apoptotic characteristics in 11-*epi*-SA-treated HA22T cells. (**A**) Detection of DNA fragments after 11-*epi*-SA treatment for 24 h at the indicated concentrations. M, marker; C, control; (**B**) Detection of apoptotic HA22T cells after 11-*epi*-SA treatment by annexin V-fluorescein isothiocyanate (FITC)/propidium iodide (PI) analysis.

Some antitumor compounds exhibit apoptotic characteristics toward cancer cells. To determine whether the antiproliferative effect of 11-*epi*-SA on the HA22T cells was due to the induction of apoptosis, we performed a DNA fragmentation assay, a semi-quantitative method for measuring apoptosis. DNA fragmentation was detectable after 11-*epi*-SA treatment for 24 h at the concentrations of 3, 6, and 9 μg/mL (Figure 4A). Next, the flow cytometric analysis was performed. In this assay, the cells were categorized into three populations through annexin V-fluorescein isothiocyanate (FITC)/propidium iodide (PI) double staining: apoptotic cells (annexin V-FITC-positive and PI-negative), necrotic cells (annexin V-FITC-positive and PI-positive), and viable cells (annexin V-FITC-negative and PI-negative) [43]. We observed that treatment with 9 μg/mL 11-*epi*-SA for 24 h resulted in a 15.6% increase in the apoptotic cell population (Figure 4B). According to the aforementioned results, the cytotoxic effect of 11-*epi*-SA on HA22T cells was through the activation of the apoptotic pathway, leading to programmed cell death.

### 2.3. Global Proteomic Profiling of 11-epi-SA-Treated HA22T Cells

According to the aforementioned results, the main cytotoxic effect of 11-*epi*-SA on HA22T cells was through the induction of apoptosis, which was manifested by apoptosis characteristics including cell shrinkage, DNA fragment formation, and mitochondrial loss (Figure 3B and Figure 4B).

**Figure 5:**
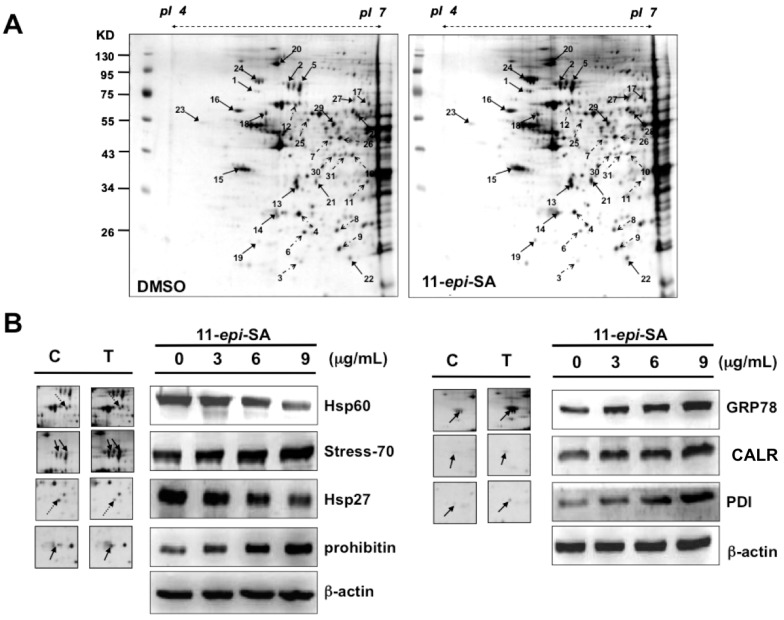
Identification of differentially expressed proteins from mock- and 11-*epi*-SA-treated HA22T hepatoma cells by two-dimensional gel electrophoresis (2DE). (**A**) HA22T cells treated with DMSO (control) or 11-*epi*-SA at the concentration of 9 μg/mL for 24 h, were harvested and cell lysates were prepared as described in the Materials and Methods section. One hundred micrograms of protein were subjected to 2DE, and the proteins were visualized by silver staining. PDQuest image analysis software (Bio-Rad) was used for detecting the differential protein spots; (**B**) Validation of identified selected proteins from 2DE. The cell lysates prepared from mock- and 11-*epi*-SA-treated cells were subjected to sodium dodecyl sulfate polyacrylamide gel electrophoresis (SDS-PAGE) for protein separation. Some identified proteins including glucose-regulated protein 78 (GRP78)/binding immunoglobulin protein (Bip), calreticulin (CALR), protein disulfide isomerase (PDI), 60 kDa heat shock protein (Hsp60), stress-70, 27 kDa heat shock protein (Hsp27), and prohibitin were detected by Western blotting by using specific antibodies as indicated. C: control, DMSO-treated cells; T: 11-*epi*-SA-treated cells. β-actin was used as the loading control.

We next attempted to obtain the pattern of differentially expressed proteins associated with 11-*epi*-SA treatment in the HA22T cells. To this end, proteomic analysis was employed for identifying differentially expressed proteins between the DMSO- and 11-*epi*-SA-treated HA22T cells. The 2DE maps of the 11-*epi*-SA-treated HA22T cells were compared with those of the DMSO-treated cells to identify the differentially expressed proteins. In total, 100 μg of protein was subjected to the 2DE (isoelectric point (pI) 4–7) and visualized by silver staining (Figure 5A). Subsequently, PDQuest image analysis software (Bio-Rad, Hercules, CA, USA) was used to detect the differential protein spots; spots demonstrating a more than 1.5-fold intensity difference (upregulation or downregulation) in the 2DE maps were selected for further protein identification by using LC–MS/MS. Table 1 lists the identified proteins with their accession numbers, MS/MS-matched sequences, MASCOT scores, molecular weight (MW)/pI values, sequence coverage, and fold of change. In total, 15 differentially expressed proteins were upregulated and 12 were downregulated upon 11-*epi*-SA treatment. These altered proteins were distributed throughout the gels, implying that multiple clusters of proteins were involved in the effects of 11-*epi*-SA on HA22T cells.

The first group involved stress response-related proteins including peroxiredoxin-2, heat-shock protein 27 (Hsp27), thioredoxin-dependent peroxide reductase, protein disulfide isomerase (PDI), protein DJ-1, calreticulin (CALR), and GRP78/Bip. The second group contained protein folding-related proteins including stress-70, 60 kDa heat-shock protein (Hsp60), and T-complex protein 1 subunit zeta. The third group comprised signal transduction pathway-related proteins including cytoplasmic protein NCK adaptor protein 1 (NCK1), guanine nucleotide-binding protein subunit beta 2, and Rho GDP-dissociation inhibitor 1. Other altered proteins included mRNA-processing proteins; heterogeneous nuclear ribonucleoprotein A/B, H1, and H3; cell differentiation protein; and vimentin. Overall, the detection of these differentially expressed proteins in the 11-*epi*-SA-treated HA22T cells suggested the role of 11-*epi*-SA in the regulation of cellular homeostasis. To validate the pattern of differentially expressed proteins, which was obtained from the 2DE maps (Figure 5A), several identified proteins were investigated by Western blotting by using anti-prohibitin, anti-CALR, anti-GRP78, anti-Hsp60, anti-stress-70, anti-Hsp27, and anti-PDI-specific antibodies. Compared with the DMSO-treated HA22T cells, five upregulated proteins (prohibitin, CALR, GRP78/Bip, stress-70, and PDI) and two downregulated proteins (Hsp60 and Hsp27) were observed in the 11-*epi*-SA-treated HA22T cells; this observation is consistent with the protein expression pattern from the 2DE maps (Figure 5B).

### 2.4. 11-epi-SA Causes Mitochondrial Dysfunction

**Figure 6:**
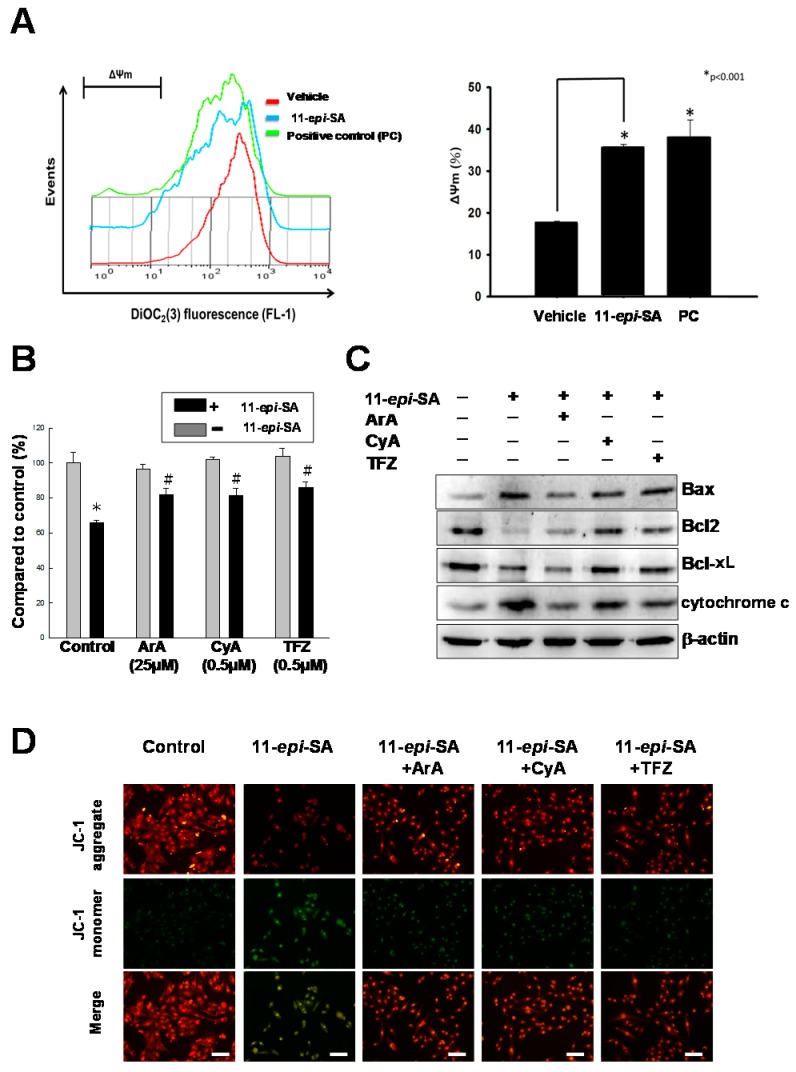
Treatment with 11-*epi*-SA induces apoptosis through the mitochondria-related pathway. (**A**) Measurement of the mitochondrial membrane permeability transition in HA22T cells after 11-*epi*-SA treatment. The quantitation of the mitochondrial membrane potential measured by 3,3′-diethyloxacarbocyanine iodide (DOC_2_(3)) fluorescence (FL-1) (**left**) and calculated as relative (ΔΨm) (**right**) from mock-, 11-*epi*-SA-treated, and 3-chlorophenylhydrazone (positive control, PC)-treated cells (* *p* < 0.01); (**B**) Effect of different mitochondrial permeability transition inhibitors on the cell viability after treatment with 11-*epi*-SA. Mock- and 11-*epi*-SA-treated cells were cultured in the presence of different inhibitors as indicated for 24 h, followed by examination of cell viability by the MTT assay. Data shown are representative of three independent experiments (* *p* < 0.01, # *p* < 0.05); (**C**) Analysis of the differential expression level of some mitochondria-related apoptotic pathway proteins in response to different inhibitors as indicated in 11-*epi*-SA-treated cells; (**D**) HA22T cells were treated as indicated, stained with 5,5,6,6-tetrachloro-1,1,3,3-tetraethylbenzimidazolcarbocyanine iodide (JC-1) dye, incubated for 20 min at 37 °C and 5% CO_2_, and imaged under a fluorescence microscope at the emission wavelengths of 580 nm (red, upper panels, JC-1 aggregate) and 530 nm (green, middle panels, JC-1 monomer). ArA, aristolochic acid; Bax, Bcl-2-associated X; Bcl-x_L_, B-cell lymphoma-extra large; Bcl-2, B-cell lymphoma 2; CyA, cyclosporin A; TFZ, trifenoperazine. Scale bar: 50 µm.

Apoptosis, or programmed cell death, plays a major role in regulating cell physiological processes to remove DNA-damaged cells with minimal damage to surrounding normal cells or tissue in addition to serving as a defense system against numerous toxic chemicals [44,45]. The presence of mitochondrial dysfunction is considered to be a feature of the apoptotic pathway, which has been thoroughly characterized. In this study, the mitochondrial membrane potential (ΔΨm) was assessed using 5,5,6,6-tetrachloro-1,1,3,3-tetraethylbenzimidazolcarbocyanine iodide (JC-1) dye to evaluate changes in the mitochondrial membrane potential. The 11-*epi*-SA-treated HA22T cells exhibited a significant reduction in the red fluorescence signals, whereas an increase in the green fluorescence signals was detected, signifying that the depletion of the mitochondrial membrane potential was due to the 11-*epi*-SA treatment (Figure 6A).

According to a previous study, the mitochondrial permeability transition underlies 11-*epi*-SA-induced mitochondrial uncoupling, which in turn leads to mitochondrial dysfunction [46]. Aristolochic acid (ArA), cyclosporin A (CyA), and trifenoperazine (TFZ) are potent mitochondrial permeability transition inhibitors. In certain hepatocytes, CyA and TFZ prevented mitochondrial depolarization to protect the cells from mitochondrial dysfunction. To further validate whether the 11-*epi*-SA-induced apoptosis in HA22T cells was caused partially by mitochondria-related depolarization, HA22T cells were pretreated with TFZ, ArA, and CyA (inhibitors of the mitochondrial permeability transition) before 11-*epi*-SA treatment. All these inhibitors protected the mitochondrial permeability transition from 11-*epi*-SA, which resulted in the restoration of cell viability (Figure 6B). In addition, several mitochondria-related apoptotic pathway proteins, including Bax, Bcl-2, Bcl-x_L_, and cytochrome *c* proteins, were investigated by Western blotting by using specific antibodies as indicators (Figure 6C).

Finally, the inhibitor-treated HA22T cells were stained with the JC-1 dye, and they were then imaged under a fluorescence microscope for examining the mitochondrial membrane potential. The inhibitors reduced the green fluorescence signals, suggesting mitochondrial permeability transition inhibition, which was induced by adding 11-*epi*-SA (Figure 6D). Overall, these results confirmed that 11-*epi*-SA induced apoptosis in the HA22T cells through the mitochondria-related pathway.

### 2.5. The Mitochondria-Related Pathway Is Activated by 11-epi-SA in a Dose and Time-Dependent Manner

**Figure 7:**
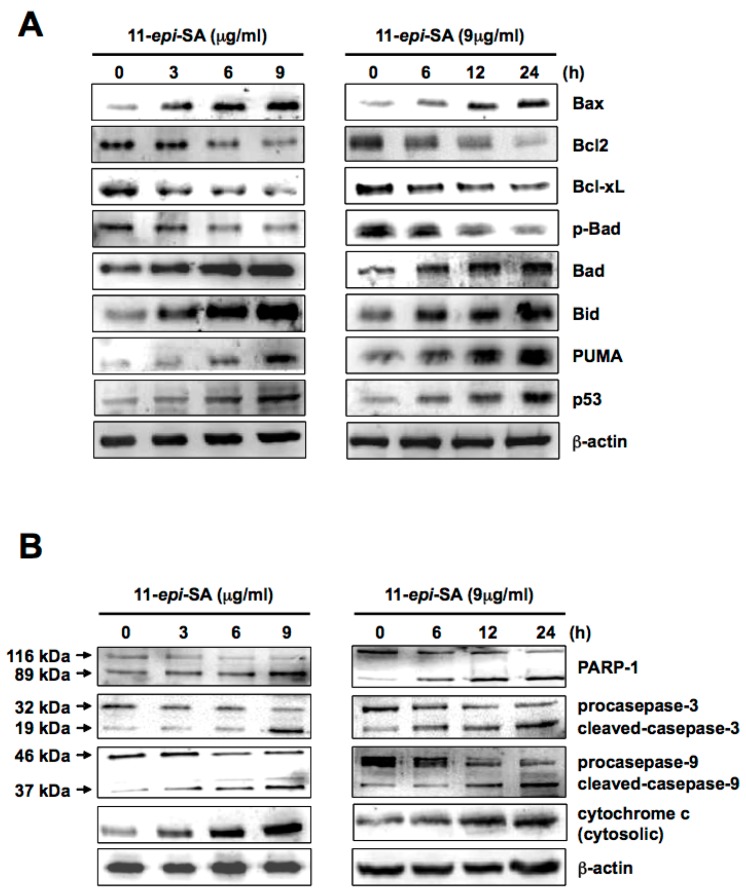
Treatment with 11-*epi*-SA activates mitochondria-related-apoptotic pathway proteins. (**A**) HA22T cells were treated without or with 11-*epi*-SA at different concentrations (**left**) and different time points (**right**) as indicated. The cells were then harvested and protein expression level was assessed by Western blotting using specific antibodies as indicated, and β-actin as loading control; (**B**) Caspase-dependent pathway proteins were activated in the 11-*epi*-SA-treated cells. The cell lysates were harvested after 11-*epi*-SA treatment as indicated above, followed by the examination of poly(ADP-ribose) polymerase 1 (PARP-1), caspase-3, -9, and cytochrome *c* proteins by using the specific antibodies as indicated. β-actin was used as the loading control.

Two features of the apoptotic pathway that cells undergo to control cell proliferation have been proposed. The caspase-dependent pathway is a thoroughly characterized apoptosis-related pathway. Bax, a proapoptotic member protein, plays a central role in mitochondria-dependent apoptosis. In particular, the protein expression ratio of Bax to Bcl2 (antiapoptotic protein) alters the mitochondrial membrane potential and initiates cytochrome *c* release and the subsequent caspase activation [47,48,49]. To acquire more specific evidence of 11-*epi*-SA-induced apoptosis through the caspase-dependent pathway, several mitochondria-related proteins, including Bax, Bcl2, Bcl-x_L_, Bad, phospho (p)-Bad, Bid, PUMA, and p53, were analyzed by Western blotting using specific antibodies as described in the Materials and Methods section. Expression levels of Bax, Bad, Bid, PUMA, and p53 increased in a dose- and time-dependent manner after 11-*epi*-SA treatment (Figure 7A). By contrast, expression levels of Bcl2, Bcl-x_L_, and phospho-Bad (p-Bad) decreased after 11-*epi*-SA treatment. A study suggested that mitochondrial cell death is mainly regulated by Bcl-2 family members [50]. The Bax and Bcl2 protein expression ratio is crucial for cytochrome *c* release as well as to ensuing caspase activation [51,52,53]. Moreover, the BH3-only proteins, including Bad, Bim, Bid, Bik, and PUMA, were immediate upstream triggers for Bax activation (directly or indirectly) through the sequestration of Bcl2 and Bcl-x_L_ proteins [29]. Bax activation is regulated by p53 through a transcription-dependent and/or transcription-independent mechanism whereas p53 functions in a similar manner as BH3-only proteins do [54]. The differential expression of the aforementioned apoptosis-related proteins suggested that 11-*epi*-SA-induced apoptosis was associated with the activation of the mitochondrial pathway in HA22T cells (Figure 7A).

### 2.6. The Caspase-Dependent Pathway Is Activated in 11-epi-SA-Treated HA22T Cells

The caspase-dependent pathway contributes to programmed cell death. Two indicator proteins, caspase 9 and 3, are activated in mitochondrial cell death signals [55,56,57,58,59]. To investigate whether caspase 3 and 9 were involved in the 11-*epi*-SA-induced apoptosis in HA22T cells, expression levels of caspase 3 and caspase 9 were examined after 11-*epi*-SA treatment. As shown in Figure 7B, both procaspase 9 and 3 expression levels were downregulated in time- and dose-dependent manners in 11-*epi*-SA-treated cells. The activation of caspase 3 was indicated by the presence of a 19 kDa proteolytic polypeptide fragment after 11-*epi*-SA treatment. Similar results were observed in caspase 9-cleaved polypeptide fragments (37 and 35 kDa, see Figure 7B). In addition, a previous study reported that PARP-1 (116 kDa) was cleaved by the caspase protein during apoptosis [60]. Our results showed that the PARP-1 cleavage fragment (89 kDa) was upregulated after 11-*epi*-SA treatment. Overall, these results revealed that mitochondrial-related apoptosis genes were activated by 11-*epi*-SA treatment.

### 2.7. 11-epi-SA Induces the Activation of the ER Stress-Induced Pathway

**Figure 8:**
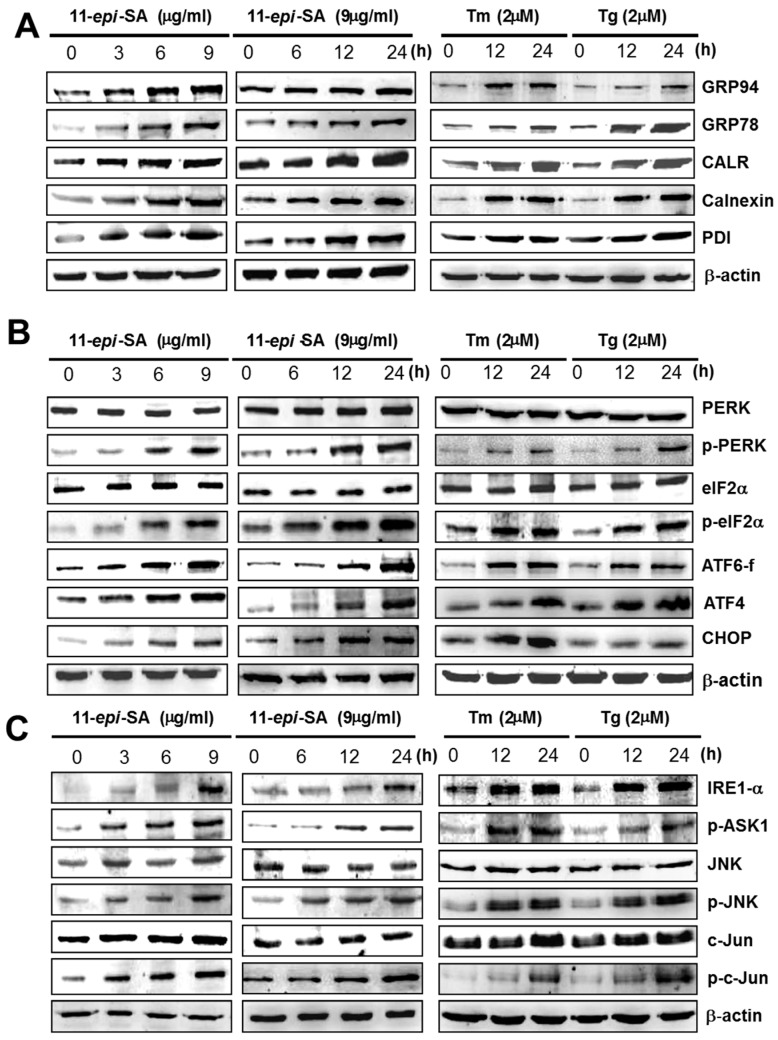
Treatment with 11-*epi*-SA stimulates the endoplasmic reticulum (ER) stress-mediated apoptotic pathway. HA22T cells were treated without or with 11-*epi*-SA at different concentrations (**left**) and different time points (**middle**) as indicated. The cells were then harvested to assess the presence ER stress-related apoptotic pathway proteins as indicated. (**A**) ER-related proteins, 94 kDa glucose-regulated protein (GRP94), GRP78/BiP, CALR, calnexin, and PDI were examined by specific antibodies; (**B**) ER stress-mediated apoptotic proteins, protein kinase R (PKR)-like endoplasmic reticulum kinase (PERK), phospho-PERK (p-PERK), eukaryotic initiation factor 2 alpha (eIF2α), p-eIF2α, activating transcription factor (ATF) 6 fragment (ATF6-f), ATF4, and CCAAT/enhancer binding protein (C/EBP) homologous protein (CHOP) were detected by specific antibodies; (**C**) Some other ER stress-related proteins, inositol-requiring enzyme 1 alpha (IRE1-α), phospho-apoptosis signal-regulating kinase 1 (p-ASK1), c-Jun N-terminal kinase (JNK), phospho-JNK (p-JNK), c-Jun and phosphor-c-Jun (p-c-Jun) were detected by specific antibodies. Please note that cells treated with tunicamycin (Tm) or thapsigargin (Tg) were considered to indicate ER stress response as shown in the **right** side of each panel.

As shown here, 11-*epi*-SA triggers cell death, typically apoptosis, possibly through the ER stress-induced pathway. The ER is the cardinal signal-transducing organelle that senses and responds to changes in homeostasis. In the lumen of the ER, the accumulation of unfolded proteins can disturb homeostasis and cause stress response, subsequently inducing destructive responses or self-rescuing in cells. Three major responses to ER stress exist: UPR, ER-associated degradation, and apoptosis [61,62]. The accumulation of unfolded proteins in the ER, such as GRP78/BiP involved in protein folding, initiates the release of transmembrane proteins PERK, IRE1-α, and activating transcription factor (ATF) 6 that can activate the UPR under ER stress. UPR can be a destructive response or a self-rescuing response depending on the elicited signaling pathway. For example, the PERK-mediated signaling pathway can enhance cell survival through autophagy [63] or cause apoptosis in cells through the upregulation of *ATF4* and the proapoptotic transcription factor *CCAAT/enhancer binding protein* (*C/EBP*) *homologous protein* (*CHOP*) [64]. In the present study, the expression levels of ER chaperones, namely GRP78/BiP, 94 kDa glucose-regulated protein (GRP94), CALR, and calnexin, were investigated by Western blotting by using specific antibodies as described in the Materials and Methods section. The results indicated that the expression levels of these ER chaperone proteins increased in dose- and time-dependent manners in response to the 11-*epi*-SA treatment (Figure 8A). Notably, 11-*epi*-SA induced ER stress response-related protein expression levels similar to those engendered by the two ER stress inducers, tunicamycin (Tm) and thapsigargin (Tg). These results implied that 11-*epi*-SA triggered apoptosis in the HA22T cells through the ER stress pathway.

PERK, ATF6, and IRE1-α belong to the ER-associated sensor proteins, which are involved in the transcriptional activation of the GRP78 promoter [65]. Under ER stress, the phosphorylated form of PERK can phosphorylate eukaryotic initiation factor 2 alpha (eIF2α), leading to reduced transcription and protein synthesis; the IRE1-α and ATF6 pathways promote the expression of the ER chaperone [66,67,68]. In this study, we investigated the aforementioned ER stress sensor proteins to validate the involvement of ER stress in 11-*epi*-SA-induced apoptosis in HA22T cells, and the results are outlined as follows. First, the expression levels of phosphorylated PERK and eIF2α increased, whereas the total protein levels of PERK and eIF2α remained unchanged (Figure 8B). Second, the expression levels of ATF6 fragment (ATF6-f) and ATF4, which represent the transcription factors of ER chaperones, increased after 11-*epi*-SA treatment (Figure 8B). Finally, the upregulated expression of CHOP, a characteristic of ER stress-mediated apoptosis, was detected in the 11-*epi*-SA-treated HA22T cells (Figure 8B). The PERK signal transduction pathway activates phospho-eIF2α (p-eIF2α), thereby increasing the expression of *ATF4*, followed by binding to the promoter of the *GRP78*/*Bip* gene, thus increasing the expression of *GRP78*/*Bip* [69,70]. ATF6 is a basic leucine zipper protein that can constitutively induce *GRP78*/*Bip* expression. Under ER stress, endogenous ATF6 (p90 ATF6) is cleaved into a 50 kDa fragment (p50 ATF6) as the transcription factor enters the nucleus and activates the UPR genes, including *GRP78/Bip* and *CALR* [71]. CHOP activation downregulates Bcl-2 protein levels, thereby increasing the expression of the BH3-only protein Bim [72]. According to our results, 11-*epi*-SA indeed triggered ER stress and induced the activation of HA22T cell death program.

Various cell signals as well as regulatory proteins control the process of apoptosis. Here, we explored other proteins involved in the apoptotic pathway, and the results are outlined as follows: (1) The expression levels of IRE1-α and phospho-apoptosis signal-regulating kinase 1 (p-ASK1) were upregulated by 11-*epi*-SA treatment; and (2) the levels of phosphorylated JNK (p-JNK) and c-Jun (p-c-Jun) increased, but those of JNK and c-Jun remained unchanged (Figure 8C). The results indicated that the expression levels of both proteins were affected in response to 11-*epi*-SA treatment. The IRE1-α protein acts as a serine–threonine kinase and an endoribonuclease. The activation of IRE1-α can trigger the binding of the adapter molecule tumor necrosis factor receptor-associated factor 2 (TNFR-2) and the recruitment of ASK1. ASK1 is a ubiquitously expressed mitogen-activated protein kinase kinase kinase (MAP3K), which can activate both JNK and p38 pathways in response to various stimuli. JNK and p38 have complex functions and modulate an extensive range of cellular effects including apoptosis, differentiation, proliferation, inflammation, and migration [73]. ASK1 has been widely accepted as a major modulator of JNK and p38 activities that regulate cell death [74]. In general, these results suggested that at least two pathways, the PERK–ATF6–CHOP and IRE1-α–JNK–cJun, were involved in the 11-*epi*-SA-induced apoptosis in the HA22T cells.

### 2.8. Inhibition of ER Stress-Related Pathways Rescues the 11-epi-SA-Induced Cytotoxicity of HA22T Cells

**Figure 9:**
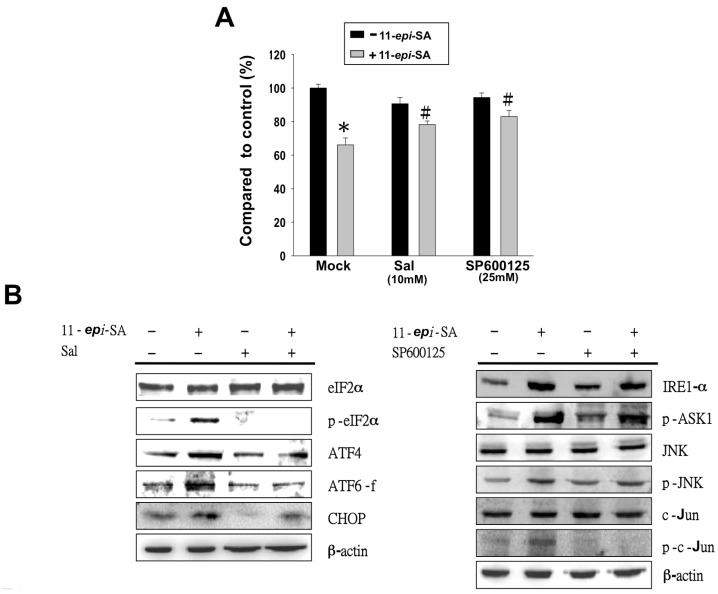
Treatment with 11-*epi*-SA induces HA22T cell apoptosis through two distinct pathways. (**A**) Two inhibitors, salubrinal (Sal) and SP600125, rescued the 11-*epi*-SA-induced cytotoxicity in HA22T cells. # *p* < 0.05; * *p* < 0.001; (**B**) Validation of altered protein expression caused by these two inhibitors. HA22T cells were treated with 11-*epi*-SA in the presence or absence of Sal (**left**) or SP600125 (**right**) for 24 h, cells were harvested and cell lysates were processed to assess ER stress-mediated pathways proteins as indicated by Western blotting using specific antibodies as indicated.

To further characterize whether 11-*epi*-SA-induced apoptosis occurs through the aforementioned ER-stress related pathways, we tested two inhibitors (salubrinal (Sal) and SP600125) to elucidate if the PERK- and IRE1-α induced apoptosis occurs through 11-*epi*-SA treatment. The results indicated that the viability of the 11-*epi*-SA-treated HA22T cells increased from 64% to 78% after treatment with Sal at a concentration of 10 mM. The 11-epi-SA-treated HA22T cells increased from 64% to 71% at a concentration of 25 mM (Figure 9A). Moreover, we observed a significant reduction of p-eIF2α in 11-epi-SA-treated HA22T cells with the addition of Sal (Figure 9B, left panel). Similarly, the reduced protein level of p-c-Jun was confirmed by Western blotting by using an anti-p-c-Jun-specific antibody in SP600125-treated cells (Figure 9B, right panel). Because Sal is an ER stress inhibitor, it can inhibit eIF2α phosphorylation and protect cells against ER stress-mediated apoptosis. SP600125 is a JNK-specific inhibitor and blocks the IRE1-α–JNK–cJun pathway to prevent cell apoptosis. Therefore, we conclude that 11-*epi*-SA induces apoptosis in HA22T cells through the PERK–ATF6–CHOP or IRE1-α–JNK–c-Jun pathway.

## 3. Discussion

The signal transduction pathways and proteins associated with the effects of such compounds can be studied by performing a comparative proteomic analysis. The data from such an analysis may provide clues for further understanding the potential mechanisms of these compounds at the molecular level. In this study, we investigated the antitumor effects of 11-*epi*-SA on HA22T cells by using MTT and wound healing assays in addition to flow cytometry. We further studied the identified proteins associated with the effect of 11-*epi*-SA because they were involved in the PERK–ATF6–CHOP and IRE1-α-induced signal pathways.

### 3.1. 11-epi-SA Induces Apoptosis in HCC Cells

Several compounds isolated from soft corals have been demonstrated to induce antiapoptotic and antitumor effects on different cancer cell lines [14,16]. A principal effect is the apoptosis-induced activity of anticancer therapies and this activity is a valuable guide for predicting tumor response after anticancer treatment. Although 11-*epi*-SA is an active compound extracted from the cultured soft coral *S. flexibilis*, the exact antitumor molecular mechanism of this compound has yet to be reported.

Apoptosis is characterized by several cellular and biochemical hallmarks including DNA chromatin condensation, nucleus fragmentation, and phosphatidylserine externalization [43,51,75,76]. In the present study, apoptosis was the major phenomenon of 11-*epi*-SA-induced cytotoxicity, in which substantial changes in apoptotic morphological characteristics were observed in HA22T cells. This apoptotic effect was further validated by the considerable increase in the apoptotic cell population and stimulation of DNA laddering after 11-*epi*-SA treatment (Figure 4).

### 3.2. Mitochondria and ER Are Involved in 11-epi-SA-Induced Cytotoxicity

Drug-based treatment of tumor cells can exert various effects on the cells. Such effects include necrosis, in which cells lose membrane integrity and die rapidly because of cell lysis. Certain drug-treated cells can stop actively growing and/or dividing, or they can trigger the genetic program of controlled cell death (apoptosis). Because proteins are the key elements for cellular activities, cellular protein expression alterations related to drug stimulation can provide valuable information for understanding the mechanism of drug activities. We thus employed a comparative proteomic approach to examine protein alterations associated with 11-*epi*-SA-induced cell apoptosis.

Prohibitin works together with chromatin remodeling molecules in transcriptional regulation [77,78,79,80], and it is also involved in p53-associated apoptosis [81,82,83]. Prohibitin also regulates cellular signaling, cell migration and cell proliferation in addition to stabilizing mitochondrial proteins. The stress-70 protein is a cellular chaperone involved in cell proliferation, differentiation, and tumorigenesis. The expression level of stress-70 protein substantially increases in bladder tumor cells on treatment with 13-acetoxysarcocrassolide, a strong suppressor of cancer cells. The Hsp60 protein facilitates the correct folding of the newly translated polypeptides, particularly those located inside the mitochondria, indicating that Hsp60 plays a role in the enhancement of cell survival under stress conditions [84]. Moreover, the Hsp60 protein interacts with the proapoptotic Bax and Bak proteins and prevents apoptosis onset. Downregulation of Hsp60 expression increased the relocalization of Bax within the mitochondrial membrane fraction [84]. The interaction between Hsp60 and Bax may be considered crucial in preventing apoptosis in normal cells [85,86,87]. A previous study reported a correlation between the downregulation of Hsp60 protein expression and the appearance of apoptosis in an oral squamous carcinoma cell line after treatment with a soft coral-isolated compound (i.e., 11-dehydrosinulariolide), indicating that the Hsp60 protein exerts antitumor effects in vitro [16]. Overall, in this study, the upregulation of stress-70 and prohibitin proteins as well as the downregulation of Hsp60 appeared to be associated with mitochondria-mediated cell cytotoxicity in 11-*epi*-SA-treated HA22T cells.

Other proteins involved in the process of protein folding, synthesis, and secretion, including GRP78/Bip, CALR, and protein disulfide-isomerase A3 (PDIA3), were also considerably upregulated by 11-*epi*-SA treatment. GRP78/Bip is a key protein in maintaining a normal ER function and protecting the ER from stress stimulation. GRP78/Bip can control the activation of transmembrane ER stress sensors through a binding-release mechanism and can activate downstream signaling pathways [88]. The altered protein expression levels of GRP78/Bip, PDI, CALR, and transitional ER ATPase also suggest that the ER system was under challenge after 11-*epi*-SA treatment in HA22T cells.

### 3.3. Mitochondrial Dysfunction Is a Crucial Event in 11-epi-SA-Related Cell Cytotoxicity

Numerous studies have reported that the mitochondria play a major role in apoptosis. In this study, the mitochondria-related apoptotic pathway was involved in 11-*epi*-SA-induced cytotoxicity in HA22T cells. In fact, 11-*epi*-SA-treated HA22T cells exhibited a decreased mitochondrial membrane potential as early as 4 h after treatment (Figure 6A). To further investigate whether mitochondrial permeabilization was involved in 11-*epi*-SA-induced apoptosis, we used the phospholipase inhibitors ArA, CyA, and TFZ to detect the effects on 11-*epi*-SA-related cytotoxicity. The results reveal that using these inhibitors partially prevented the cytotoxic effects of 11-*epi*-SA on HA22T cells (Figure 6B).

Evidence shows that the abnormal expression of Bcl-2 and the overexpression of caspase-3 result in defective apoptosis, implying that the Bcl-2 gene is an anti-apoptotic gene. In this study, the results indicate that the expression of the Bcl-2 protein decreased on 11-*epi*-SA treatment, signifying that Bcl-2 family proteins served as the central regulators of mitochondrial integrity. Under normal conditions, Bcl-2 and Bcl-x_L_, which are antiapoptotic members of the Bcl-2 family, form heterodimers with the proapoptotic element Bax. By contrast, under the mitochondrial stress induced by 11-*epi*-SA treatment in HA22T cells used in this study, the insertion of Bax into the mitochondrial outer membrane increased membrane permeability, subsequently leading to the release of cytochrome *c* through the pores on the outer mitochondrial membrane [89]. Similarly, the Bad protein, a proapoptotic member of the Bcl-2 family, promotes cell death by displacing Bax from binding to the Bcl-2 and Bcl-x_L_ proteins [90,91]. The phosphorylated form of Bad promotes the binding of Bad to the 14-3-3 protein to prevent an association between Bad and Bcl-2 or Bcl-x_L_ [91]. Therefore, in this study, the downregulation of Bcl-2, Bcl-x_L_, and phosphorylated Bad in addition to the upregulation of Bad, Bax, Bid, and PUMA on 11-*epi*-SA treatment demonstrated the antitumor effect of 11-*epi*-SA on HA22T cells. Additionally, we observed the release of proapoptotic mitochondrial factors such as cytochrome *c*, which activate caspase-9 to form an apoptosome complex, as well as the activation of caspase-3 and cleavage of PARP-1 to execute apoptosis. Overall, these results show that mitochondrial dysfunction may, at least partially, contribute to the induction of cytotoxicity by 11-*epi*-SA treatment in HA22T cells.

### 3.4. ER Stress Partially Initiates 11-epi-SA-Induced Cytotoxicity in HA22T Cells

Two main apoptotic pathways exist: the extrinsic or death receptor pathway and the intrinsic or mitochondrial pathway. In addition, many reports have indicated that both pathways are linked, and several proteins in one pathway can influence those in the other pathway [92]. Among the cellular compartments, the ER is involved in the intrinsic apoptotic pathway, CALR–calnexin and GRP78/Bip–GRP94 belong to two chaperone systems in the ER, in which CALR and calnexin are the chaperone lectins and GRP78/Bip and GRP94 are the chaperones of the heat-shock protein family. These two systems have distinct functions: CALR–calnexin is responsible for the newly synthesized glycoproteins, and GRP78/Bip–GRP94 plays a major role in the folding and maturation of non-glycosylated proteins. In this study, the two systems exhibited similar responses to the ER stress induced by 11-*epi*-SA treatment in HA22T cells.

We also examined the ER stress sensor proteins to confirm the involvement of ER stress in apoptosis. We detected phosphorylated forms of both PERK and eIF2α proteins indicating that 11-*epi*-SA treated HA22T cells induced apoptosis through the ER stress-relative pathway. Similarly, the upregulation of ATF4 was a response to the ER stress exerted on 11-*epi*-SA-treated cells, and the ATF6 protein was cleaved to its active form, ATF6-f (Figure 8B). These signal transduction pathways were the adaptation responses to the ER stress, and they suppressed protein synthesis and then released the burden of ER stress, subsequently increasing the transcription of ER-related chaperone genes. Moreover, in response to ER stress, ATF6 translocated from the ER to the Golgi, where it was cleaved. The cleaved form of ATF6 then entered the nucleus and served as a transcription factor for GRP78/Bip. Our results indicated that ATF6, PERK and eIF2α proteins were responsible for the 11-*epi*-SA-induced apoptotic process through the ER stress pathway in HA22T cells.

Finally, when the ER stress was prolonged and became severe, these responses triggered apoptosis. Under physiological conditions, CHOP was expressed at low levels, but it was strongly expressed in response to the ER stress. The overexpression of CHOP could lead to growth arrest and apoptosis, as revealed by the study on traditional ER stress inducers (Tm and Tg). Sal is a selective inhibitor of eIF2α phosphatase enzymes. This observation indicates that the ER stress partially contributed to the 11-*epi*-SA-induced apoptosis in HA22T cells.

## 4. Materials and Methods

### 4.1. Reagents

Many reagents, including Dulbecco’s modified Eagle’s medium (DMEM), trypsin-ethylenediaminetetraacetic acid, fetal bovine serum (FBS), and phosphate-buffered saline (PBS), were obtained from Biowest (Nuaillé, France). The 2-D Quant Kit protein assay kit, immobilized pH gradient (IPG) buffer, and isoelectrofocusing strips were obtained from GE Healthcare (Buckinghamshire, UK). Polyvinylidene difluoride (PVDF) membranes, Suicide Track DNA ladder isolation kit, and goat anti-rabbit and horseradish peroxidase-conjugated immunoglobulin (Ig) G were obtained from Millipore (Bellerica, MA, USA). Protease inhibitor cocktail, DMSO, Sal, CyA, ArA, TFZ, MTT, Tm, Tg, and rabbit anti-human β-actin antibodies were obtained from Sigma (St. Louis, MO, USA). Cell extraction buffer was obtained from BioSource International (Camarillo, CA, USA). An annexin V-FITC/PI apoptosis detection kit was obtained from Pharmingen (San Diego, CA, USA). JC-1 cationic dye fluorescence kit was obtained from Biotium (Hayward, CA, USA). The enhanced chemiluminescence (ECL) Western blotting reagents were obtained from Pierce Biotechnology (Rockford, IL, USA). A 4′-6-diamidino-2-phenylindole (DAPI) fluorescence kit and DeadEnd Fluorometric terminal deoxynucleotidyl transferase dUTP nick end labeling (TUNEL) fluorescence kit were obtained from Promega (Madison, WI, USA). Antibodies against prohibitin, p53, Hsp60, Hsp27, stress-70, eIF2α, and glucose-related protein 94 (GRP94) were obtained from Epitomics (Burlingame, CA, USA). Antibodies against CALR, calnexin, protein disulfide-isomerase (PDI), glucose-related protein 78 (GRP78), and cleaved-ATF6 and ATF4 were obtained from ProteinTech Group (Chicago, IL, USA). Antibodies against caspase 3, caspase 9, cytochrome *c*, Bid, Bax, Bad, p-Bad, PUMA, CHOP, IRE1-α, PERK, phospho-PERK (p-PERK), p-eIF2α, p-ASK1, JNK, p-JNK, c-Jun, p-c-Jun, Bcl-2, and Bcl-x_L_ were obtained from Cell Signaling Technology (Danvers, MA, USA).

### 4.2. Cell Culture and Drug Treatment

Human hepatoma cell lines HA22T, HepG2, Hep3B, and Huh7 were obtained from the Bioresource Collection and Research Center (Food Industry Research and Development Institute, Hsin Chu, Taiwan). Cells were cultured in DMEM with 1% penicillin or streptomycin (10,000 U·mL^−1^ penicillin and 10 mg·mL^−1^ streptomycin) and 10% (*v*/*v*) FBS supplemented with 4 mM l-glutamine and 1 mM sodium pyruvate in a humidified atmosphere with 5% CO_2_ at 37 °C. Control cultures were prepared by adding DMSO at the same final concentration as in the treated samples (0.01% *v*/*v*). Cells were treated with various concentrations of 11-*epi*-SA (1, 3, 5, 9, and 12 μg/mL) and harvested after 24 h of incubation. To determine whether 11-*epi*-SA induced cell cytotoxicity through ER stress or mitochondria transmembrane potential depletion, an ER stress inhibitor, 10 μM Sal, and the mitochondrial permeability transition inhibitors, 0.5 μM CyA, 25 μM ArA, and 0.5 μM TFZ, were used to pretreat cells 1 h before 11-*epi*-SA treatment.

### 4.3. Cell Viability Assay

The effects of 11-*epi*-SA on the viability of HA22T, HepG2, Hep3B, and Huh7 cell lines were investigated using the MTT assay. Cells (1 × 10^4^/well) were incubated in 96-well plates. After treatment with several concentrations of 11-*epi*-SA as indicated, the cells were then further incubated for 4 h at 37 °C. To solubilize purple-blue MTT formazan crystals in viable cells, 200 μL of DMSO was added to each well. Finally, the absorbance was monitored using a microtiter plate enzyme-linked immunosorbent assay (ELISA) reader at a wavelength of 595 nm. All experiments were repeated three times under the same conditions.

### 4.4. Wound Healing and Trans-Well Migration Assays

For the wound healing assay, HA22T cells were seeded in six-well plates (8 × 10^5^/well). After the cells reached confluence, a scratch or wound was made with a pipette tip in each well. Unattached tumor cells were washed with PBS and refreshed with FBS-containing medium. Images of the control and experimental groups (0, 3, 6, and 9 μg/mL 11-*epi*-SA) were acquired at 0, 24, and 48 h after treatment. HA22T cells with or without 11-*epi*-SA treatment were assayed for cell migration as described [40].

### 4.5. Flow Cytometric Assessment of Apoptosis

HA22T cells (2 × 10^5^ cells/mL) were cultured in 6cm Petri dishes and incubated for 24 h. Apoptotic processes induced by 12 h 11-*epi*-SA treatment were determined using the annexin V-FITC/PI apoptosis detection kit according to manufacturer’s instructions on a FACScan flow cytometer (Becton-Dickinson, Mansfield, MA, USA). Data analysis was performed with Cell-Quest software (Becton-Dickinson). The cells in the FITC-positive and PI-negative fractions were defined as apoptotic cells.

### 4.6. DNA Fragmentation Assay

Total DNA from HA22T cells treated with 1% DMSO and 3, 9, and 12 μg·mL^−1^ 11-*epi*-SA for 12 and 24 h was extracted using the Suicide Track DNA ladder isolation kit. In total, 20 μg of DNA were analyzed on 2% agarose gels in tris/borate/ethylenediaminetetraacetic acid (EDTA) (TBE) buffer. Agarose gels were run at 50 V for 120 min. Bands were detected by ethidium bromide staining, and they were visualized under UV light (260 nm).

### 4.7. Mitochondrial Transmembrane Potential Analysis

Changes in mitochondrial membrane potential were measured by flow cytometry by using a cationic cyanine fluorescence dye DiOC_2_(3) (MitoProbe, Invitrogen, Carlsbad, CA, USA) according to the manufacturer’s protocol. Briefly, 11-*epi*-SA-treated cells were washed and resuspended with 1 mL of PBS, followed by incubation with 50 nM of DiOC_2_(3) for 30 min at 37 °C in the dark. Cells treated with a mitochondrial uncoupling agent, 3-chlorophenylhydrazone (CCCP), served as a positive control. Data were analyzed using FlowJo software [41].

### 4.8. Protein Preparation and Measurement

HA22T cells were treated with different concentrations of 11-*epi*-SA (0, 3, 6, and 9 μg·mL^−1^) for 24 h or left untreated, and then lysed with the protease inhibitor cocktail and cell extraction buffer. All proteins in the supernatant were then precipitated overnight (−20 °C) by using three times the volume of 10% trichloroacetic acid/acetone solution containing 20 mM dithiothreitol (DTT). The pellets were collected and resuspended overnight in a rehydration buffer (6 M urea, 2 M thiourea, 0.5% IPG buffer, 20 mM DTT, 0.5% 3-[(3-cholamidopropyl)dimethylammonio]-1-propanesulfonate (CHAPS), and 0.002% bromophenol blue) at 4 °C. The protein concentration was determined using the 2-D Quant Kit (GE Healthcare).

### 4.9. Two-Dimensional Gel Electrophoresis and Protein Identification by Liquid Chromatography–Tandem Mass Spectrometry

In this study, 2DE was performed with the GE Healthcare Ettan IPGphor 3 and SE 600 Ruby electrophoresis unit (Hoefer, Holliston, MA, USA) by using a previously described protocol [93]. A sample was dissolved in the rehydration buffer as described earlier and applied on an IPG strip in a strip holder. Proteins (100 μg) extracted from whole cells were loaded on an 11 cm IPG strip (pI 4–7, Immobiline DryStrip), and subsequently separated by SDS-PAGE (12.5%). 2DE images were acquired in triplicate for each sample and normalized before statistical analysis.

### 4.10. Immunofluorescence Microscopy

DAPI and TUNEL assays in addition to cationic dye JC-1 staining were performed according to the procedures described in a previous study [93]. HA22T cells (1 × 10^5^ cells/well) were seeded in a 12-well plate and treated with different concentrations of 11-*epi*-SA (0, 3, 6, and 9 μg/mL) and then grown in 5% CO_2_ at 37 °C. Next, cells in each treatment group as well as control, non-treated cells were fixed with 4% paraformaldehyde in PBS for 15 min and stained with DAPI, DeadEnd Fluorometric TUNEL System, and cationic dye JC-1 according to the manufacturer’s instructions. Cells were photographed under a fluorescence microscope (Olympus IX71 CTS, Chinetek Scientific Limited, Kowloon, Hong Kong, China).

### 4.11. Western Blotting

Proteins were separated by SDS-PAGE (12.5%) and transferred to PVDF membranes (Millipore) for 1.5 h at 400 mA by using TE 22 transfer tank (Hoefer). The membranes were then incubated overnight with different primary antibodies at 4 °C, washed three times with 10 mM NaH_2_PO_4_, 130 mM NaCl, 0.05% Tween-20 (PBST), and then probed with the secondary antibody (i.e., horseradish peroxidase conjugate, 1:5000 in blocking solution) for 1 h. After the membrane washing process, the immunoreactive bands were visualized through chemiluminescence by adding ECL Western blotting reagents (Pierce Biotechnology). β-actin was used as a loading control.

### 4.12. Statistical Analysis

Results were pooled from three independent experiments. Data from the MTT and cell migration assays as well as the flow cytometric analysis were subjected to Student’s *t* test (Sigma-Stat 2.0, San Rafael, CA, USA). Results with *p* < 0.05 were considered statistically significant.

## 5. Conclusions

**Figure 10:**
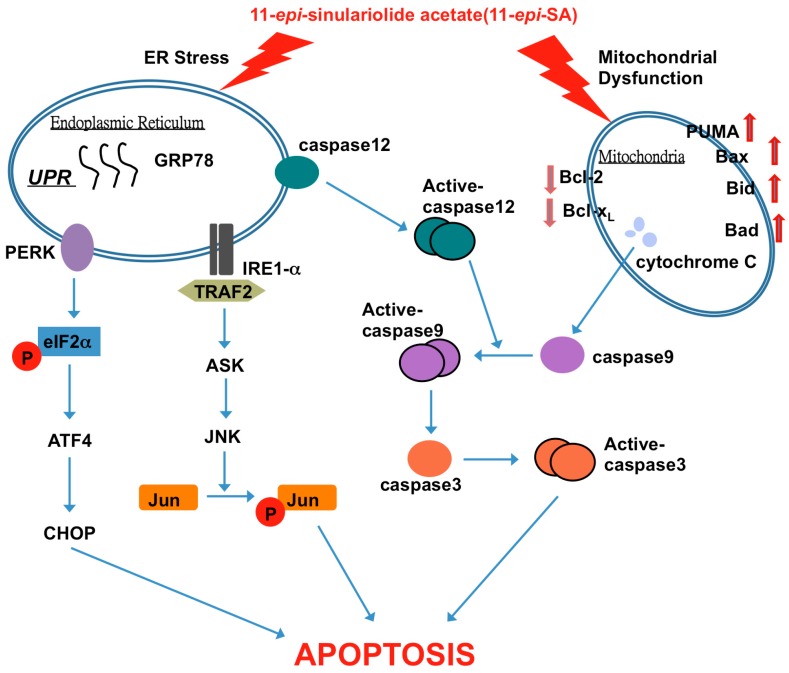
11-*epi*-SA-induced apoptotic pathway in HA22T cancer cells. The anticancer effect of 11-*epi*-SA is mediated by the induction of mitochondrial dysfunction and the ER stress signaling pathway. TRAF2, tumor necrosis factor receptor-associated factor 2.

In this study, we investigated and confirmed that 11-*epi*-SA can induce apoptosis in HA22T cells. Proteomic analysis of the total protein profiles indicates that proteins related to energy metabolism and protein synthesis were involved in the apoptotic process. Additional functional studies demonstrate that 11-*epi*-SA induced mitochondrial dysfunction and the ER stress system (Figure 10). This is the first report describing the effect of 11-*epi*-SA on ER stress and mitochondrial dysfunction. Our findings also demonstrate that 11-*epi*-SA induced cytotoxicity in HA22T cells through multiple apoptosis-inducing pathways, suggesting that 11-*epi*-SA can be a potent anticancer drug for cancer treatment. Additional and more detailed functional studies will be performed to identify the specific targets of 11-*epi*-SA in tumor cells.

## Figures and Tables

**Table 1:** Protein identification by liquid chromatography–tandem mass spectrometry (LC-MS/MS).

Spot No.	Protein Name	Accession No.	Calculated MW/pI	Peptide Matched	Sequence Covered %	MASCOT Score	Regulation (Fold Change)
1	Lamin-A/C	P02545	74.09/6.57	20	22	126	+3.3
2	Stress-70	P38646	73.65/5.87	54	46	570	+2.5
3	Peroxiredoxin-2	P32119	21.87/5.66	31	43	316	−2.3
4	Isopentenyl-diphosphate delta-isomerase 2	Q9BXS1	26.7/6.01	1	5	75	−2.5
5	Stress-70	P38646	73.63/5.87	100	63	1233	+3.1
6	Heat-shock protein 27	P04792	22.76/5.98	52	71	753	−3.9
7	Cytoplasmic protein NCK1	P16333	42.83/6.06	6	9	78	−2.8
8	Heat-shock protein 27	P04792	22.76/5.98	90	72	945	−2.4
9	Thioredoxin-dependent peroxide reductase	P30048	27.67/7.67	20	35	209	−3.0
10	Heterogeneous nuclear ribonucleoprotein A/B	Q99729	36.59/9.04	3	9	72	−3.2
11	Heterogeneous nuclear ribonucleoprotein H3	P31942	36.91/6.37	14	30	173	−2.9
12	60 kDa heat-shock protein	P10809	61.01/5.7	13	11	70	−1.8
13	Guanine nucleotide-binding protein G(I)/G(S)/G(T) subunit beta 2	P62879	37.32/5.6	13	22	52	+3.1
14	Prohibitin	P35232	29.78/5.57	385	88	10,621	+2.2
15	Nucleophosmin	P06748	32.55/4.64	6	22	107	+2.1
16	Protein disulfide-isomerase	P07237	57.08/4.76	51	52	314	+2.7
17	T-complex protein 1 subunit zeta	P40227	57.98/6.23	27	37	278	+1.8
18	Vimentin	P08670	53.61/5.06	52	52	507	+2.1
19	Rho GDP-dissociation inhibitor 1	P52565	23.19/5.02	6	15	67	+2.6
20	Transitional endoplasmic reticulum ATPase	P55072	89.22/5.14	55	47	594	+4.4
21	l-lactate dehydrogenase B chain	P07195	36.61/5.71	16	32	147	+3.1
22	Protein DJ-1	Q99497	19.87/6.33	58	62	368	+2.0
23	Calreticulin	P27797	48.11/4.29	28	43	193	+1.9
24	78 kDa glucose-regulated protein precursor	P11021	72.28/5.07	61	56	900	+5.2
25	Tubulin alpha-ubiquitous chain	P68363	50.12/4.94	23	35	251	−1.8
26	Ornithine aminotransferase	P04181	48.85/6.57	21	56	1065	−1.9
27	T-complex protein 1 subunit beta	P78371	57.45/6.01	33	40	386	+2.1
28	Succinyl-CoA:3-ketoacid-coenzyme A transferase 1	P55809	56.12/7.14	14	12	234	−2.4
29	Heterogeneous nuclear ribonucleoprotein H	P31943	49.19/5.89	45	32	626	+1.7
30	Macrophage capping protein	P40121	38.49/5.88	17	29	283	−2.3
31	Macrophage capping protein	P40121	38.49/5.88	3	6	72	−1.9

MW/pI, molecular weight/isoelectric point.

## Supplementary Materials

Supplementary materials can be found at www.mdpi.com/1422-0067/17/11/1787/s1.
