# Supplementary material for: Cytotoxicity of 11-epi-Sinulariolide Acetate Isolated from Cultured Soft Corals on HA22T Cells through the Endoplasmic Reticulum Stress Pathway and Mitochondrial Dysfunction
Source: Int J Mol Sci. 2016 Oct 27;17(11):1787. doi: 10.3390/ijms17111787 (PMC5133788; doi:10.3390/ijms17111787)
Supplement: Supplementary file 1 [file ijms-17-01787-s001.pdf]

Supplementary Materials: Cytotoxicity of 11-*epi*-Sinulariolide Acetate Isolated from Cultured Soft Corals on HA22T Cells through the Endoplasmic Reticulum Stress Pathway and Mitochondrial Dysfunction

Jen-Jie Lin, Robert Y. L. Wang, Jiing-Chuan Chen, Chien-Chih Chiu, Ming-Hui Liao and Yu-Jen Wu

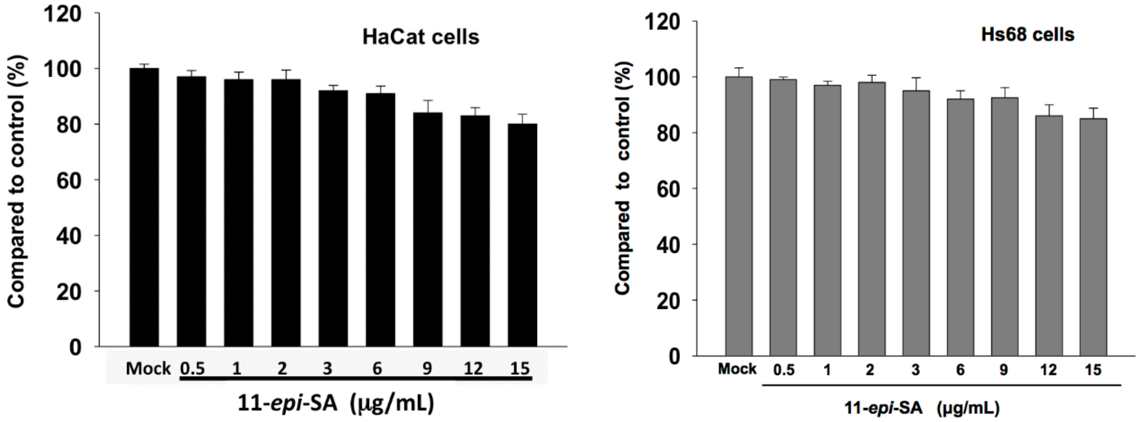

(**A**) (**B**)

Figure S1. Analysis of the cytotoxic effect of 11-*epi*-sinulariolide acetate (11-*epi*-SA) on non-hepatoma cell lines. The HaCat cells (human immortalized keratinocytes) (**A**) and Hs68 (human foreskin-derived primary cell line) (**B**) were treated with dimethyl sulfoxide (DMSO) (mock control) or various concentrations of 11-*epi*-SA for 24 h, followed by examination of cell viability by using the 3-(4,5-dimethylthiazol-2-yl)-2,5-diphenyltetrazolium bromide (MTT) assay as described in the Materials and Methods section. The 11-*epi*-SA compound exerts no cytotoxic effect on either of these cell lines.
